# Expanding the potential soil carbon sink: unraveling carbon sequestration accessory genes in vermicompost phages

**DOI:** 10.1128/aem.00296-25

**Published:** 2025-03-14

**Authors:** Shujian Yuan, Yunling Wu, Jose Luis Balcazar, Danrui Wang, Dong Zhu, Mao Ye, Mingming Sun, Feng Hu

**Affiliations:** 1 Soil Ecology Lab, Jiangsu Provincial Key Laboratory of Coastal Saline Soil Resources Utilization and Ecological Conservation, Jiangsu Collaborative Innovation Center for Solid Organic Waste Resource Utilization & Jiangsu Key Laboratory for Solid Organic Waste Utilization, Nanjing Agricultural University 70578 , Nanjing, China; 2 Catalan Institute for Water Research (ICRA), Girona, Spain; 3 University of Girona 16738 , Girona, Spain; 4 Zhejiang Key Laboratory of Urban Environmental Processes and Pollution Control, Ningbo Urban Environment Observation and Research Station, Chinese Academy of Sciences, Ningbo, China; 5 Key Laboratory of Soil Environment and Pollution Remediation, Institute of Soil Science, Chinese Academy of Sciences 229806 , Nanjing, China; Colorado School of Mines, Golden, Colorado, USA

**Keywords:** vermicompost, carbon sequestration, phage AMGs, soil

## Abstract

The compost microbiome is important in regulating soil carbon sequestration. However, there is limited information concerning phage communities and phage-encoded auxiliary metabolic genes (AMGs) in compost-applied soils. We combined metagenomics and meta-viromes to explore the potential role of bacterial and phage communities in carbon sequestration in the compost microbiome. The experiment comprised swine manure compost (SW) and vermicompost (VE) applied to the soil along with a control treatment (CK). The bacterial community richness decreased after swine manure application and increased after vermicomposting compared to the control treatment. The phage community in the vermicompost-applied soil was dominated (63.1%) by temperate phages. In comparison, the communities of the swine manure compost-applied soil (92.7%) and control treatments (75.4%) were dominated by virulent phages. Phage-encoded carbon sequestration AMGs were detected in all three treatments, with significant enrichment in the vermicompost-applied soil. The average carbon sequestration potential (the coverage ratio of phage AMGs:total genes) of phage AMGs (*aceF*, *GT*11, and *GT*6) in the vermicompost-applied soil (65.18%) was greater than in the swine manure-applied (0) and control soils (50.21%). The results highlight the role of phage-encoded AMGs in improving soil carbon sequestration in vermicompost-applied soil. The findings provide new avenues for increasing soil carbon sequestration.

**IMPORTANCE:**

The phage-bacteria interactions have a significant impact on the global carbon cycle. Soil microbial carbon sequestration is a process in combination withcarbon sequestration genes and growth activity. This is the first study aimed at understanding the carbon sequestration potential of phage communities in vermicompost. The results of this study provide variations in carbon sequestration genes in vermicompost microbial communities, and some novel phage auxiliary metabolic genes were revealed to assist bacterial communities to increase soil carbon sequestration potential. Our results highlight the importance of phages in soil carbon sequestration from the perspective of phage-bacterial community interactions.

## INTRODUCTION

Phages are the most abundant biological entities on Earth, and they are widely distributed in the soil (1, 2). The number of phages in terrestrial ecosystems usually exceeds that of bacteria, with up to 10^10^ phage-like particles per gram of soil (3). Based on their life history, phages are classified as virulent or temperate phages (4). Virulent phages complete the reproductive cycle by lysing host bacterial cells and promote carbon mineralization through “viral shunting,” whereas temperate phages replicate along with their hosts without lysing the host cells (5). Soil virulent phages act as controllers of host bacterial population densities and can lead to host bacterial lysis in a short time. This process releases large amounts of organic matter that can be taken up and utilized by neighboring microbes, thereby promoting soil nutrient cycling and rapid turnover of the microbial community (6–8). In addition, there is increasing evidence that phages can provide an adaptive advantage to host bacteria by carrying auxiliary metabolic genes (AMGs) that complement the host metabolism or introduce new metabolic functions during infection (4, 9).

Phage-encoded AMGs participate in multiple processes, including element cycling, pollutant degradation, energy metabolism, functioning as enzyme cofactors, and photosynthesis (10–15). Also, phages have an important regulatory role in the soil carbon cycle (16). Soil phages encode genes for glycosyltransferases (GTs), polysaccharide hydrolysis, polysaccharide binding, and glycoside hydrolase (16–20). In addition, the phage-encoded glycosyltransferases *GT*75 and *GT*2 were identified in soil microbiomes; these enzymes are associated with the biosynthesis of cell membrane components and play critical roles in promoting soil carbon sequestration (17). However, the effect of different manure compost applications on the functional type and metabolic potential of phage-carrying carbon-associated AMGs in agricultural soils remains less well understood.

Soil carbon sequestration is important for mitigating global climate change and dealing with carbon emissions (21). With changes in agricultural practices, organic fertilizers are often applied to agricultural soils as a substitute for chemical fertilizers, and the most popular choices include vermicompost and swine manure (22). These compost fertilizers regulate soil nutrient cycling, organic matter decomposition, and the capacity for carbon cycling by affecting the microbial community. The impact of microbes on the soil carbon cycle is primarily related to carbon sequestration and decomposition. Microbes promote carbon sequestration by directly fixing atmospheric CO_2_ or synthesizing difficult-to-decompose organic matter (23, 24). Meanwhile, microbes secrete a variety of enzymes to degrade soil organic matter and thereby accelerate the soil carbon cycle (25). The application of swine manure compost and vermicompost has been shown to promote soil organic carbon (SOC) storage (26, 27). However, the impacts of changes in the phage community and phage-encoded AMGs on carbon sequestration in swine manure compost-applied soil (SW) and vermicompost-applied soil (VE) have not yet been investigated. It is important to further explore the role played by microbial communities in the soil carbon balance after the application of different forms of compost manure, especially the role played by the phage community.

In this study, we hypothesized that the phage community in vermicompost would encode more carbon sequestration-related AMGs compared to swine manure in order to assist bacteria in collaboratively maintaining the soil carbon balance. We used metagenome and meta-virome sequencing analyses to explore the changes in the bacterial community composition and the functions of phage-carrying auxiliary metabolic genes in soils applied with different organic fertilizers to analyze the effects of bacteria and phages individually on the soil carbon cycle and to elaborate the mechanism of phages in assisting the bacteria to maintain the soil carbon balance. Our findings provide a novel perspective for using vermicompost to optimize land management practices and enhance the carbon sink potential of agricultural soils using bacterium-phage interactions.

## MATERIALS AND METHODS

### Soil collection

The soil samples were collected from farmland soils with long histories of different compost applications in Nanjing, Jiangsu Province (118°47′01″E, 31°43′33″N). Three different soils were collected, including control soil (CK) with no fertilizer application, swine manure application, and vermicompost manure application. Swine manure compost and vermicompost were both applied at 3.0t/ha in spring and fall for 10 consecutive years (2012–2022). The soil samples were collected from a depth of 15 cm–20 cm from five evenly distributed points in the field, mixed into a composite sample, stored at 4°C, and sent back to the laboratory for analysis. The physicochemical properties of the soils are listed in Table S1.

### Metagenome analysis of soil samples

Soil DNA extraction and metagenome sequencing analysis were carried out according to the method of Zheng et al. (13), with detailed information provided in the supplemental text. Briefly, total DNA was extracted from 0.5 g of fresh soil samples following the manufacturer’s instructions using a FastDNA Spin kit for soil (MP Biomedicals). Its purity and concentration were determined by a Life Technologies Qubit 4.0 (Table S2). Sequencing libraries were prepared using an NEB Next Ultra DNA Library Prep Kit for Illumina (New England Biolabs) according to the protocol, and their quality was assessed using a Qubit dsDNA HS Assay Kit and Agilent 4200 system. Nine libraries for whole-genome shotgun sequencing were constructed, and paired-end (2 × 150 bp) sequencing was performed based on an Illumina HiSeq 2500. After quality control of the raw data using Cutadapt (v.1.2.1), clean reads were obtained (Table S3) and used for *de novo* assembly using MEGAHIT. Open reading frames were predicted by MetaGeneMark, and redundant genes were removed by CD-HIT. Non-redundant unigene sequences were compared against the NCBI-nr database using DIAMOND (*e*-value ≤0.0001), and species annotation was performed using the lowest common ancestor algorithm (Table S4) (28). Microbial taxonomic alpha and beta diversity analyses were carried out using the vegan and ggplot2 packages in R. Subsequently, unigenes were mapped to clean data. The “BWA-MEM” algorithm was used to calculate the reads per kilobase per million mapped reads of genes in each sample and thereby calculate the relative gene abundance. Functional genes were annotated by comparing them with the KEGG and eggNOG databases using DIAMOND (Table S5). Additionally, genes related to carbon metabolism were further identified based on the CAZy database and classified into three categories: carbon sequestration, carbon transformation, and carbon decomposition (Table S6) (29).

### Phage DNA extraction and virome analysis

Soil phage DNA extraction and virome analysis were performed according to the method described by Zheng et al. (13; see supplemental text for detailed methods). Briefly, the total soil phage DNA was extracted following the methods described by Zheng et al. (13) and Tang et al. (30). To enrich the free phages, the soil samples (500 g) were sieved, mixed with 500 mL of 1% (wt/wt) potassium citrate buffer, incubated, and sonicated. The supernatant was centrifuged, filtered through 0.45 µm and 0.22 µm filters, and concentrated using a tangential flow filtration system (100 kDa) to obtain the free phage suspension. Soil prophages were induced by mitomycin-C solution (1 µg/mL). After incubation and shaking, the prophage solution was concentrated following the above phage extraction procedures. No contamination of bacterial DNA was found in the mixture of free phage and prophage enrichment solution, as determined by 16S rRNA gene PCR analysis. A Takara MiniBEST Viral RNA/DNA Extraction Kit 5.0 was used to extract the total phage DNA. The whole phage genome was amplified and sequenced on an Illumina Novaseq 6000 platform, generating 150 bp paired-end reads. After quality control of the raw data with Cutadapt (v.1.2.1), ~0.27 billion clean reads were obtained (Table S3).

All the clean reads were used for *de novo* co-assembly of phage sequences using MEGAHIT. Assembled contigs longer than 5,000 bp were defined as phages if they met one of the criteria (see the supplemental material). The clean reads were mapped to the phage contigs using BWA with default parameters to determine the relative abundance of phage contigs (Table S7) (31). vConTACT2 (v.2.0) was used to analyze the protein-sharing network of the phage contigs (32) and PhaGCN2.0 was used to annotate the phage family (Table S7) (33). The lifestyle of each phage contig was predicted by Deephage (Table S7) (34). A phage phylogeny was constructed using ViPTree (v.4.0) and visualized using Chiplot (35, 36). The top 10 phage families with the highest phage relative abundances, except for those that were unclassified, were selected for visualization, and their diversity was calculated using the vegan and ggplot2 packages in R.

### Phage-bacterium relationship analysis

Two methods (CRISPR spacer match and genome homology match) were used to predict the linkage between phage and bacterial sequences (Table S8). Specifically, (i) a CRISPR identification tool (CRISPRCasTyper, v 1.8.0) was used to search for bacterial spacer sequences, which were compared with phage sequences using BLASTn (v.2.13.0+) with thresholds of ≤1 mismatch, 100% identity, and 100% coverage (37); and (ii) phage genome homology matches were identified by searching against bacterial genomes using BLASTn with parameters of identity ≥90% and hit length ≥1,000 bp. Phage-host information obtained by the two methods was combined to determine the phage-host range (38). The species annotation of all the host bacterial contigs was performed using the contig annotation tool (CAT v.6.0.1) with the default parameters.

### Phage auxiliary metabolism gene identification

The identification of carbon cycle-related AMGs and other metabolic function AMGs encoded by phages was performed using VIBRANT (v.1.2.0, with default parameters) and DRAM-v (v.1.2.0) (39, 40). The AMGs were filtered according to the AMG flag type provided by DRAM-v. The above AMG data are shown in Tables S9 and S10. Non-metric multidimensional scaling (NMDS) analysis was used to detect differences in the similarity of AMGs across treatments.

The linear discriminant analysis (LDA) effect size (LEfSe) method, via Bioincloud (https://bioincloud.tech/), was used to detect biomarker AMGs that differed significantly in relative abundance between different treatments. Phyre2 was used to analyze the tertiary protein structures of the carbohydrate metabolism-associated enriched AMGs (*aceF*, *GT11*, *GT6*, *wbpD*, and *ugd*) based on the LEfSe analysis (41). The hallmark proteins of phage contigs (Table S11) were identified using the PhaVIP pipeline in PhaBOX (42). The promoters and terminators of phage contigs were recognized by BPROM (linear discriminant function [LDF] >2.75) and FindTerm (score < –12.0), respectively.

### Calculation of phage carbon sequestration potential

The metagenomic reads and virome reads were mapped against the carbon metabolism-associated bacterial genes and phage AMGs using BWA (v.0.7.17), respectively (31). The coverage for each gene was calculated using “pileup.sh” within BBMap tools (https://github.com/BioInfoTools/BBMap/blob/master/sh/pileup.sh). The phage:total gene coverage ratio was calculated by adding the phage and bacterial gene coverage values and using the sum to divide the summed phage gene coverage values (10). The phage:total gene ratio was used to estimate the contributions of phage AMGs to carbon metabolism. The cumulative phage carbon sequestration potential was determined by summing the phage:total gene coverage ratio of all sequestration-related AMGs (*aceF*, *glmS*, *gltA*, *GT*11, *GT*2, *GT*4, and *GT*6). The phage:total gene ratios were calculated as follows (using AMGs *GT*6 as an example):


$$phage\;:\;total\;\;gene\;\;ratio=\;\frac{phage\;\;AMGs\;GT6\;coverage}{phage\;AMGs\;GT6\;coverage+bacterial\;GT6\;coverage}$$

### Data statistical analysis

Data statistics in this study were performed using GraphPad Prism 8.0 (https://www.graphpad.com/) and R (v.4.4.2) (https://www.r-project.org/). The data visualization was performed using https://www.chiplot.online/ and GraphPad Prism 8.0. The alpha and beta diversities of microbial community were calculated using the vegan and ggplot2 packages in R.

## RESULTS

### Overview of soil bacterial communities

**Fig 1:**
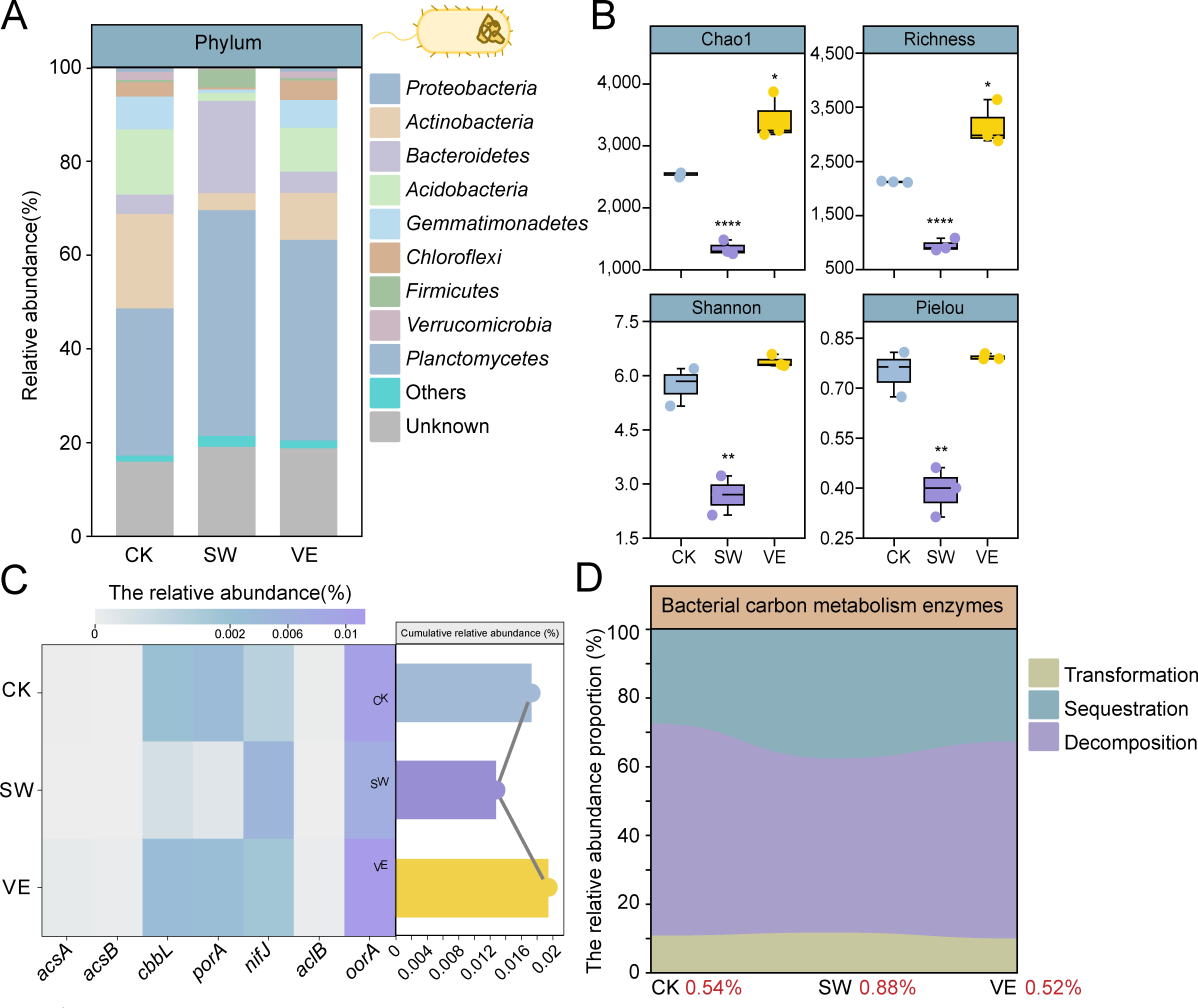
Differences in bacterial communities and carbohydrate metabolism genes in control and compost-applied soils. (A) Relative abundance of the top 10 abundant bacteria phylum classification in control (CK) and compost-applied soils (swine manure: SW; vermicompost: VE). “Others” represent the rest of the taxa. (B) Differences in bacterial taxa alpha diversity in control and compost-applied soils. (C) The heatmap on the left shows the relative abundance of bacterial carbon sequestration genes annotated by KEGG databases in control (CK) and compost-applied soils (swine manure: SW; vermicompost: VE). The bar plot on the right shows the cumulative relative abundance of carbon sequestration genes. (D) The relative abundance of bacterial carbohydrate metabolism-related enzymes annotated based on the CAZy database in control (CK) and composted soils (swine manure: SW; vermicompost: VE). The red numbers beside the group name represent the cumulative relative abundance of carbon metabolism enzymes.

To investigate the impact of organic fertilization on bacterial communities in the soils, we recovered 24,941, 24,063, and 25,221 bacterial contigs from the control soil, swine manure compost-applied soil, and vermicompost-applied soil, samples, respectively. Control and vermicompost-applied soils were dominated by *Proteobacteria* (CK: 31.36%, VE: 42.84%), *Actinobacteria* (CK: 20.14%, VE: 10.00%), and *Acidobacteria* (CK: 13.90%, VE: 9.35%; Fig. 1A). In swine manure compost-applied soil, *Proteobacteria* also occupied a dominant ecological niche with a relative abundance of 48.14%, followed by *Bacteroidetes* (19.65%) and *Firmicutes* (3.85%; Fig. 1A). Compared to the control soil, both the evenness (Pielou’s index) and the diversity (Chao1, richness, and Shannon indices) of the bacterial community in swine manure compost-applied soil were significantly decreased (*P* < 0.05; one-way analysis of variance [ANOVA]), while the diversity (Chao1 and Richness indices) of the bacterial community was significantly increased in vermicompost-applied soil (*P* < 0.05; one-way ANOVA; Fig. 1B). NMDS analysis also showed clear variation between control soil, swine manure compost-applied soil, and vermicompost-applied soil (Fig. S1). Taken together, the results suggest that vermicompost application increased the bacterial community diversity but did not change the evenness of the bacterial community or the dominant soil bacterial phyla, whereas swine manure compost-applied soil decreased the evenness and the diversity of the bacterial community and caused changes in the dominant soil bacterial phyla.

We investigated the functional classification and abundance of bacterial genes in different treatments. Based on the KEGG database, Global and overview maps (CK: 10.70%, SW: 12.12%, VE: 10.59%), Amino acid metabolism (CK: 3.31%, SW: 3.50%, VE: 3.22%), Carbohydrate metabolism (CK: 3.07%, SW: 3.07%, VE: 2.93%), and Metabolism of cofactors and vitamins (CK: 2.09%, SW: 2.82%, VE: 2.15%) were the most abundant functional categories across all the three treatments (Fig. S2). The NMDS analysis indicated variance in genetic functional categories across the samples, suggesting that swine manure compost-applied soil and vermicompost-applied soil altered the bacterial functional gene composition of the treatments (Fig. S3a). Using the KEGG and CAZy database annotations, the genes associated with bacterial community carbon sequestration, carbon transformation, and carbon decomposition in the different treatments were specifically explored. The relative abundance of the carbon sequestration genes *oorA* (CK: 0.010%; SW: 0.007%; VE: 0.011%) was the highest in all three treatments, followed by *nifJ* (CK: 0.001%; SW: 0.005%; VE: 0.002%), *porA* (CK: 0.004%; SW: 0.0003%; VE: 0.003%), and *cbbL* (CK: 0.002%; SW: 0.0006%; VE: 0.003%). The cumulative relative abundances of all carbon sequestration genes (*cbbL*, *acsA*, *acsB*, *oorA*, *nifJ*, *porA*, and *aclB*) were 0.017%, 0.013%, and 0.019% in control soil, swine manure compost-applied soil, and vermicompost-applied soil, respectively (Fig. 1C). The proportions of the carbon sequestration enzymes were 27.42%, 37.64%, and 32.68% for the control, swine manure compost-applied soil, and vermicompost-applied soil, respectively (Fig. 1D). The proportions of carbon transformation enzymes were 10.97%, 11.67%, and 9.93% for the control, swine manure compost-applied soil, and vermicompost-applied soil, respectively (Fig. 1D). Carbon decomposition enzymes accounted for the highest proportion among the detected carbon metabolism enzymes, at 61.61%, 50.69%, and 57.39% for the control, swine manure compost-applied soil, and vermicompost-applied soil, respectively (Fig. 1D). Based on the NMDS analysis, swine manure compost-applied soil and control soil showed clear variance in the composition of the carbon metabolism enzymes, while vermicompost-applied soil and control soil overlapped (Fig. S3b), indicating a similar composition of the carbon metabolism enzymes between vermicompost-applied and control soils. Thus, swine manure application altered the composition and abundance of carbon metabolism enzymes in the soil bacterial community, whereas vermicompost maintained a similar composition and abundance of carbon metabolism enzymes in the bacterial community compared to the control soil.

### Overview of the soil phage communities

**Fig 2:**
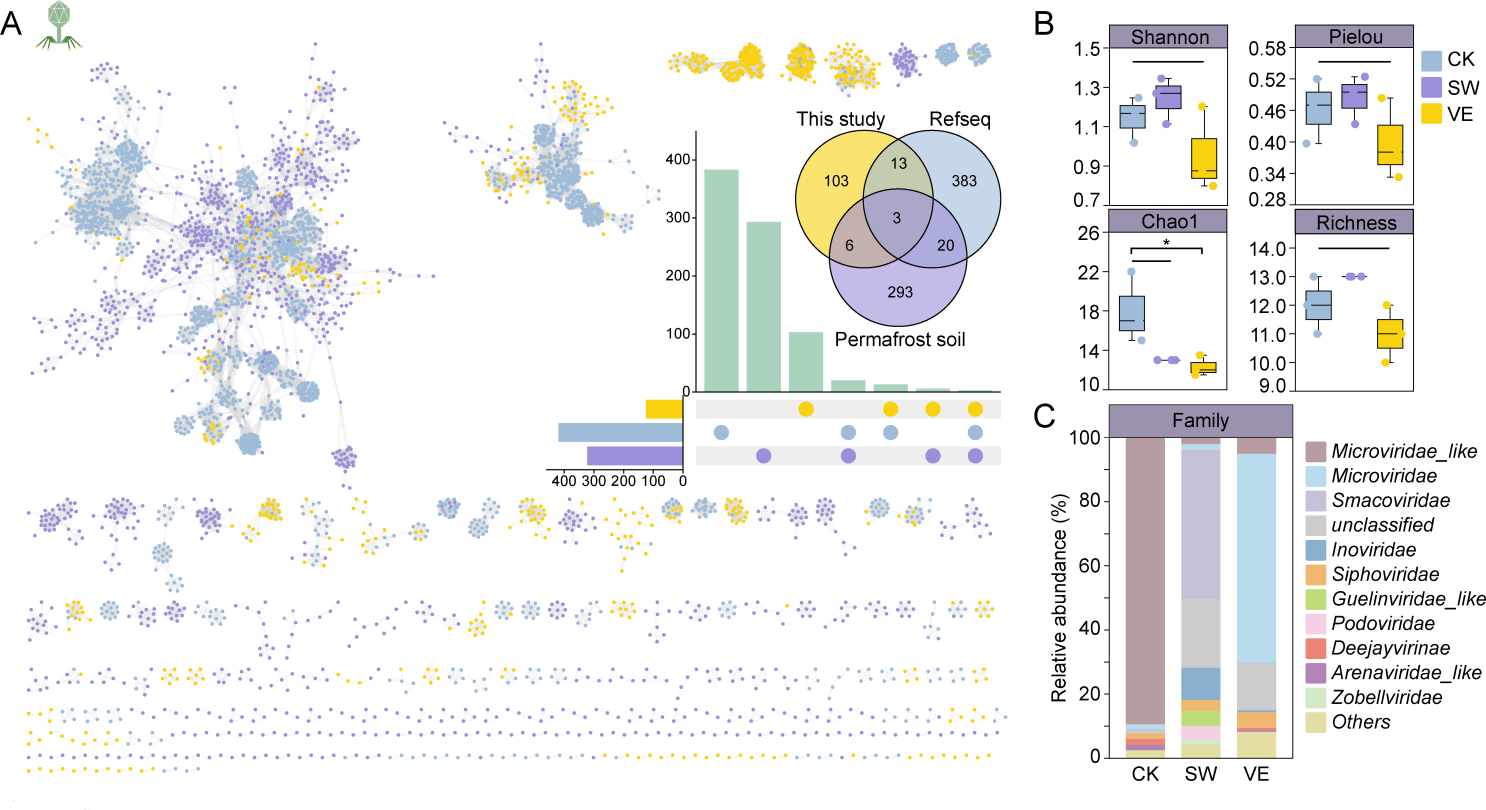
Differences in viral communities in control and composted soils. (A) Gene sharing networks of phage contigs isolated from control and manure soil (yellow), NCBI RefSeq databases (blue), and permafrost soil databases (purple). Nodes (circles) represent contigs of the viral genome, and edges indicate shared protein content. The Venn diagram and up-set analysis at the top right corner show shared and unique VCs generated by vConTACT 2 between control and manure soil, permafrost soil, and RefSeq sequences. (B) Relative abundance of the top 10 abundant viral families in control (CK) and composted soils (swine manure: SW; vermicompost: VE). “Others” represent the rest of the taxa. (C) Differences in viral family alpha diversity in control and composted soils.

To investigate the roles of the two types of manure compost on the soil phage community, we used meta-virome analysis to quantify the diversity and composition of the phage communities. Totals of 535, 658, and 485 phage contigs (>5 kb) were obtained from the meta-virome analyses of control soil, swine manure compost-applied soil, and vermicompost-applied soil, respectively. Protein similarity analysis indicated that the phage clusters from this work, permafrost soil, and RefSeq database comprised 125, 322, and 419 phage clusters, respectively (Fig. 2A). Notably, the viromes in this study shared only 16 virus clusters (VCs) with the RefSeq database, indicating that currently published phages only cover a small proportion of the phages in the compost soil (Fig. 2A). No significant changes were observed in the evenness (Pielou’s index) or abundance (Chao1, richness, and Shannon indices) of the phage community between swine manure compost-applied soil, vermicompost-applied soil, and the control soil (*P* > 0.05, one-way ANOVA; Fig. 2B), indicating that swine manure and vermicompost application did not alter the alpha diversity of the phage community.

The composition of the phage community was also investigated via analyzing the variance in the dominant phage families and the beta diversity of the phage communities across the three treatments. The top 3 dominant phage families in the control soil were *Microviridae_like* (89.40%), *Deejayvirinae* (1.92%), and *Arenaviridae_like* (1.72%). Swine manure compost-applied soil (S) was dominated by *Smacoviridae* (46.45%), *Inoviridae* (10.03%), and *Guelinviridae_like* (4.67%); the most abundant phage families in vermicompost-applied soil (E) were *Microviridae* (65.18%), *Microviridae_like* (5.00%), and *Siphoviridae* (4.77%; Fig. 2C). The beta diversity of phage communities showed distinct characteristics between the control, swine manure compost-applied soil, and vermicompost-applied soil (Fig. S4). Despite the insignificant change in the alpha diversity of phage communities, both swine manure and vermicompost application clearly affected the dominant phage families and the community structure of the soils.

### Putative phage-host linkages in control and composted soil

**Fig 3:**
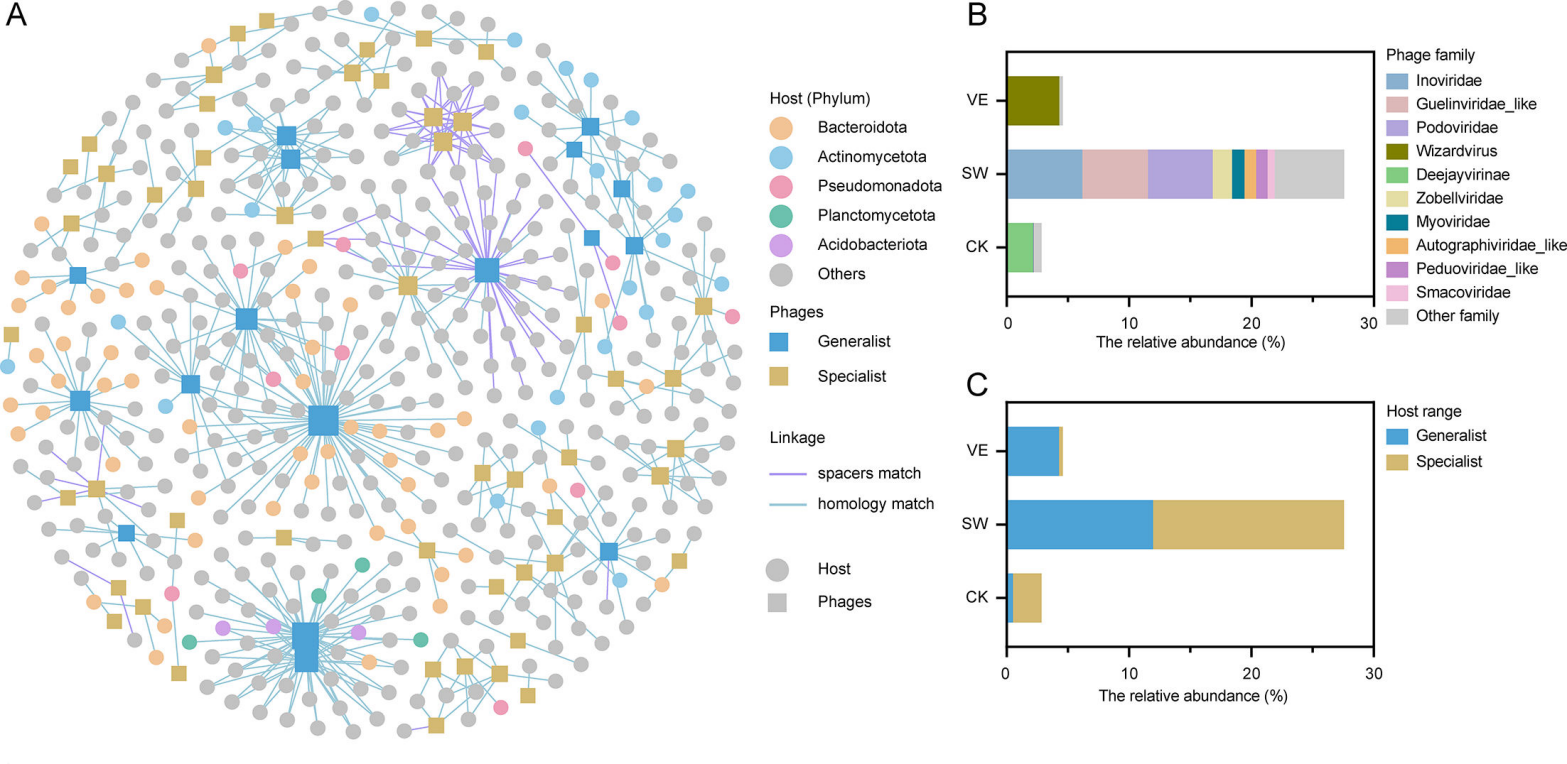
Virus-host associations in control and composted soils. (A) Phage-host bacterial associations based on CRISPR spacer and homology matches in control and composted soils. The circle colors indicate different bacterial phylum. The brown squares indicate phages that infected only a single host bacterial taxon (specialist phages), and the blue squares indicate phages that infected various host bacterial taxa (generalist phages). The lines between phages and bacteria with different colors indicate the method of identifying phage-host associations. (B) Relative abundance of phage families of phages infecting host bacteria in control and composted soils. (C) Relative abundance of specialist and generalist phages in control and composted soils.

To explore the interactions between phages and bacteria across the manure treatments, putative phage-host linkage analysis was performed via CRISPR spacer match and genome homology match approaches. A total of 69 phage contigs were linked to 447 bacterial contigs belonging to 44 known bacterial taxa, and generalist phages accounted for 24.63% (17 of 69 phage contigs; Tables S7 and S8). The dominant host bacterial phyla were *Bacteroidota*, *Actinomycetota*, *Pseudomonadota*, and *Acidobacteriota* (Fig. 3A). The dominant phages infecting the host bacteria in the control treatment, swine manure compost-applied soil, and vermicompost-applied soil belonged to *Deejayvirinae* (relative abundance: 2.04%), *Inoviridae* (relative abundance: 6.17%), and *Wizardvirus* (relative abundance: 4.19%), respectively (Fig. 3B). Additionally, the relative abundance of generalist phages in the control treatment, swine manure compost-applied soil, and vermicompost-applied soil was 0.51%, 11.96%, and 4.27%, respectively (Fig. 3C). The relative abundance of specialist phages was 2.35%, 15.62%, and 0.31% for the control, swine manure compost-applied, and vermicompost-applied soils, respectively (Fig. 3C). Overall, the phages that infected the host bacteria in swine manure compost-applied soil comprised a higher relative abundance of both generalist and specialist phages than in the control treatment and vermicompost-applied soil.

### Phage-encoded AMGs involved in carbon sequestration

**Fig 4:**
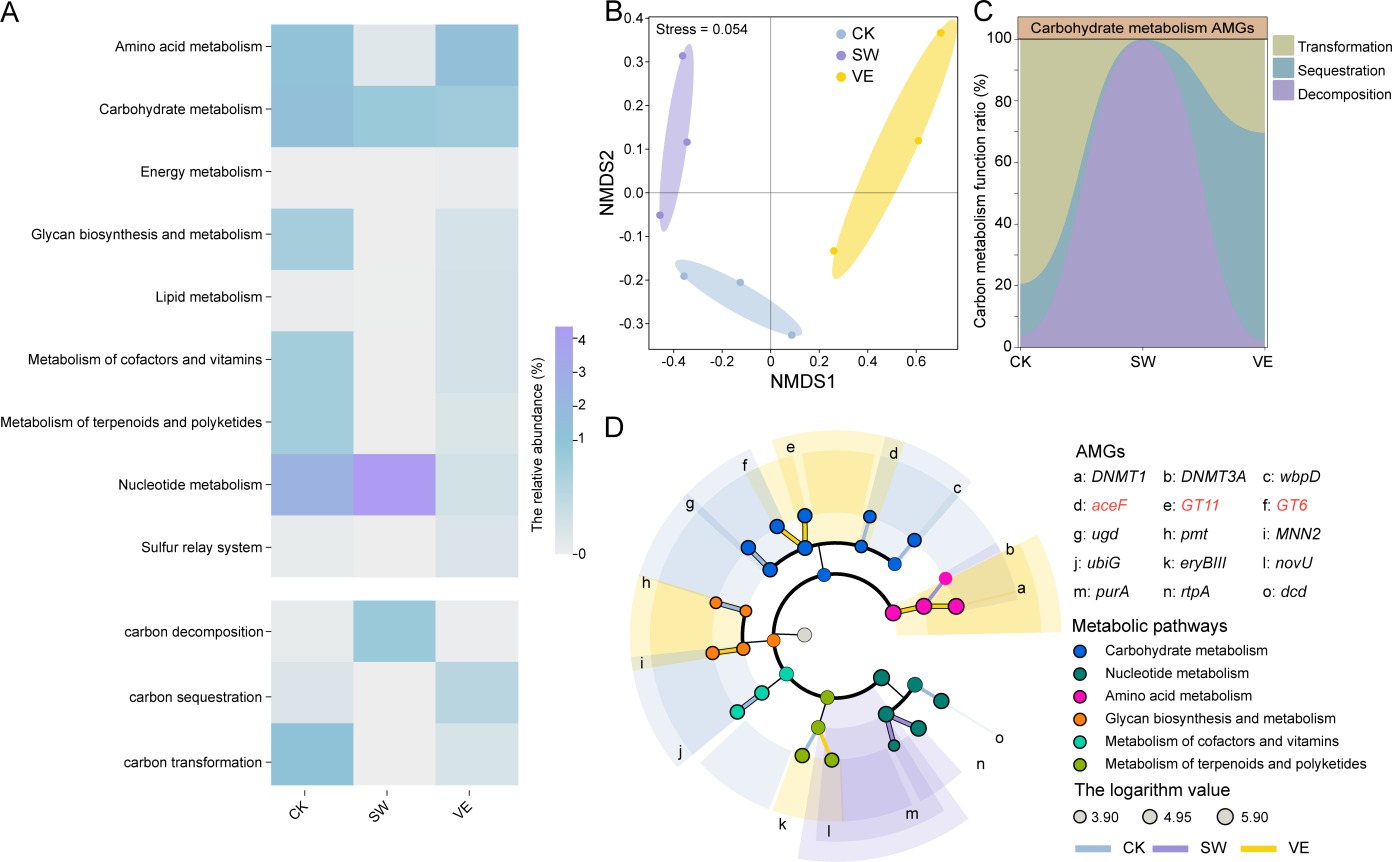
Profile of virus-encoded AMGs in control and composted soils. (A) Heatmap of the relative abundance of viral AMGs annotated by KEGG and CAZy databases in control (CK) and composted soils (swine manure: SW; vermicompost: VE). (B) NMDS analysis of viral AMGs in control and composted soils. (C) The relative abundance of AMGs for viral carbon sequestration, carbon transformation, and carbon decomposition in the different treatments. (D) LDA effect size analysis comparison of viral AMG abundance of control and composted soils (LDA score threshold >2; only clearly sorted AMGs are shown). Branch colors correspond to different treatments: blue, control (CK); purple, swine manure (SW); and yellow, vermicompost (VE). Node colors represent different AMG metabolic pathways. Viral AMG names are in the legend on the right of the dendritic diagram, with the red-lettered AMGs involved in carbon sequestration. The node size represents the logarithm value of the relative abundance of AMGs in each treatment.

To investigate the role of phages in mediating bacterial-oriented carbon cycling, we used VIBRANT and DRAM-v to identify phage-encoded AMGs across different samples. A total of 75 phage contigs encoding 102 AMGs were identified from the three treatments (Fig. S5a; Table S10). The vermicompost-applied soil contained 11 unique (i.e., exclusively present in vermicompost) phage contigs encoding AMGs, more than in swine manure compost-applied soil (five phage contigs) and the control treatment (four phage contigs; Fig. S5a). Based on the functional classification of 102 AMGs, Nucleotide metabolism (CK: 2.46%, SW: 4.34%, VE: 0.28%), Carbohydrate metabolism (CK: 1.40%, SW: 0.89%, VE: 0.82%), Amino acid metabolism (CK: 1.18%, SW: 0.14%, VE: 1.42%), and Metabolism of cofactors and vitamins (CK: 0.79%, SW: 0.0003%, VE: 0.27%) were the most abundant functions across the three treatments (Fig. 4A). In addition, there were exclusive AMGs (i.e., absent in the other two treatments) in the three groups. The two exclusive AMGs in the control treatment were involved in Carbohydrate metabolism (*pgl*) and Glycan biosynthesis and metabolism (*pmt*), and the five exclusive AMGs in vermicompost-applied soil were involved in Carbohydrate metabolism (*glmS* and *GT2*), Glycan biosynthesis and metabolism (*kdsD* and *MNN2*), and Metabolism of cofactors and vitamins (*bchE*; Fig. S5b). NMDS analysis also showed clear variance in the composition of phage AMGs between groups (Stress = 0.054; Fig. 4B), indicating potential functional differences in the phage-encoded AMGs between vermicompost-applied and swine manure compost-applied soils.

Among microbial carbohydrate metabolism-related enzymes, glycoside hydrolases (GHs), polysaccharide lyases (PLs), carbohydrate esterases (CEs), and auxiliary active enzymes (AAs) are associated with carbon decomposition; glycosyltransferases (GTs) are associated with carbon sequestration, and the transformations of different forms of organic carbon with the same number of carbon atoms were defined as carbon transformations (29). Therefore, the carbon metabolism AMGs were classified into three functional categories: carbon sequestration, carbon transformation, and carbon decomposition (Fig. 4C). Notably, the carbon metabolism-associated AMGs in the control soil, swine manure compost-applied soil, and vermicompost-applied soil were primarily involved in carbon transformation (82.48%), carbon decomposition (99.61%), and carbon sequestration (67.52%), respectively (Fig. 4C), indicating the greater potential of phage AMGs in vermicompost-applied soil for carbon sequestration than the other treatments. The carbon transformation AMGs in the control treatment were *rfbE*, *wbpD*, *ugd*, and *pgl*, the carbon decomposition AMGs in the swine manure compost-applied soil included *pel*, *CE4*, and *GH16,* and the carbon sequestration AMGs in the vermicompost-applied soil consisted of *GT6*, *GT11*, *GT4*, *GT2*, *gltA*, and *glmS* (Table S10). Overall, the number of phage AMGs involved in host bacterial carbon sequestration in vermicompost-applied soil was greater than those in swine manure compost-applied soil and the control treatment.

**Fig 5:**
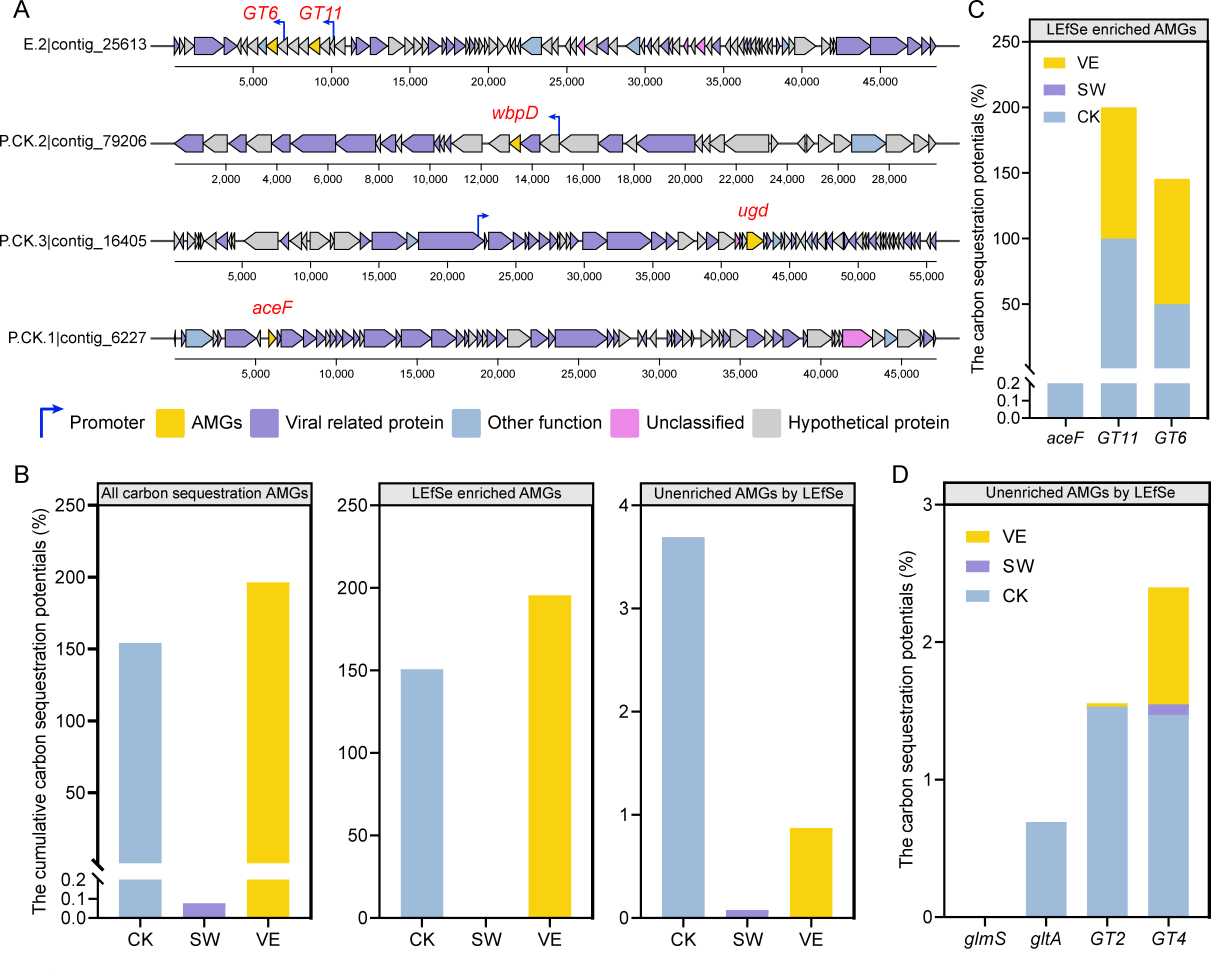
Metabolic potential of virus-encoded carbon sequestration-associated AMGs in control and composted soils. (A) Gene cluster analysis of carbon sequestration-associated AMGs, with blue arrows representing promoters in the viral contigs. (B) The cumulative carbon sequestration potentials of all carbon sequestration AMGs, LEfSe-enriched carbon sequestration AMGs (*GT11*, *GT6*, and *aceF*), and non-LEfSe-enriched carbon sequestration AMGs (*glmS*, *gltA*, *GT2*, and *GT4*) in control (CK) and composted soils (swine manure: SW; vermicompost: VE). (C) The carbon sequestration potentials of three LEfSe-enriched carbon sequestration AMGs *GT11*, *GT6*, and *aceF* in control (CK) and composted soils (swine manure: SW; vermicompost: VE). (D) The carbon sequestration potentials of four carbon sequestration AMGs *glmS*, *gltA*, *GT2*, and *GT4* unenriched by LEfSe in control (CK) and composted soils (swine manure: SW; vermicompost: VE). The contribution of phage AMGs from different soils is shown in different colors.

We performed a LEfSe enrichment analysis of phage-encoded AMGs, and a higher number of carbohydrate metabolism-associated AMGs (five AMGs, *ace*F, *ugd*, *wbp*D, *GT*11, and *GT*6) was enriched than other metabolic pathways, indicating the promising role of phage AMGs in host bacterial carbon metabolism (Fig. 4D). More specifically, the carbon sequestration *ace*F and *GT*11 and *GT*6 (glycosyltransferases belonging to the GTs family) were only enriched in the control and vermicompost-applied soils (LDA >2; Fig. 4D), indicating the promising role of vermicompost phage AMGs in carbon sequestration. In contrast, none of the above five AMGs were enriched in swine manure samples. We further investigated the expression of five enriched carbohydrate metabolism AMGs by LEfSe analysis. We first analyzed the presence of promoters in the upstream regions of these AMGs to determine their potential levels of genetic expression. The carbon sequestration-associated genes *GT*6 and *GT*11 and the carbon transformation-associated *wbp*D and *ugd* had complete promoters that were required for expression, with the only exception being the carbon sequestering AMG *ace*F that did not have a promoter in its upstream region (Fig. 5A). In addition, all five AMGs had reliable predicted tertiary protein structures (confidence >98.0%), suggesting the potential of these phage AMGs for redirecting bacterial-driven carbon metabolism (Fig. S6). In conclusion, the phage-encoded carbon sequestration AMGs *GT*6 and *GT*11 were enriched in vermicompost-applied soil, and these genes had the potential to aid host bacteria in fixing carbon.

### Carbon sequestration potential of the phage AMGs in soils

To estimate the carbon sequestration potential of the phage AMGs, we calculated the ratio of phage AMGs:total genes for each carbon sequestration AMG across the soil treatments (10). The carbon sequestration potential of phage AMGs represents the contribution of phage AMGs to carbon sequestration pathways containing the AMG across the microbial community. The carbon sequestration potential was calculated by adding up all the carbon sequestration-associated AMGs and bacterial gene coverage values and using the result to divide the coverage values of the carbon sequestration-associated phage AMGs. We first calculated the overall carbon sequestration potential of all the detected phage AMGs associated with carbon sequestration in the three treatments. The highest cumulative carbon sequestration potentials were 196.4% in vermicompost-applied soils, followed by 154.3% in the control, and 0.08% in swine manure-applied soils (Fig. 5B; Table S12).

We further classified the detected carbon sequestration-associated phage AMGs into two groups, the three enriched AMGs (*GT11*, *GT6*, and *aceF*) and the remaining AMGs that were not enriched (*glmS*, *gltA*, *GT2*, and *GT4*) by LEfSe analysis, and compared their respective carbon sequestration potentials. The cumulative carbon sequestration potentials of the AMGs (*GT*11, *GT*6, and *aceF*) were 150.6%, 0, and 195.6% for the control, swine manure-applied, and vermicompost-applied soils, respectively (Fig. 5B). Notably, *GT*11 (CK: 100.0%; VE: 100.0%) and *GT*6 (CK: 50.0%; VE: 95.6%) were the primary drivers of carbon sequestration in both vermicompost-applied and control soils (Fig. 5C). For the remaining AMGs, the cumulative carbon sequestration potentials of the AMGs *glmS*, *gltA*, *GT*2, and *GT*4 were as low as 3.7%, 0.1%, and 0.9% for control, swine manure-applied, and vermicompost-applied soils, respectively (Fig. 5B). The carbon sequestration potential of *GT*4 (CK, 1.5%; SW, 0.08%; VE, 0.9%) was the highest among the four carbon sequestration AMGs unenriched by LEfSe analysis (Fig. 5D). In summary, the potential of the phage-encoded AMGs *GT*11 and *GT*6 in vermicompost-applied soil to promote host bacterial carbon sequestration was higher than in the swine manure-applied soil and control treatments.

## DISCUSSION

### Vermicompost application increased the carbon sequestration ability of the soil bacterial community

The soil bacterial community influences the global carbon cycle, and hence climate change, by fixing atmospheric CO_2_ and synthesizing undegradable organic carbon for carbon sequestration (23, 24). The bacterial community fixes CO_2_ primarily through the Calvin cycle, the reductive tricarboxylic acid cycle, and the reductive acetyl-CoA pathway (23). In this study, the relative abundance of carbon sequestration genes was higher in the vermicompost-applied soil than in the control treatment and the swine manure compost-applied soil. In addition, the key gene *oorA* of the reductive tricarboxylic acid cycle was the most abundant carbon sequestration gene in vermicompost-applied soils. The bacteria fixing and utilizing CO_2_ through the reductive tricarboxylic acid cycle are commonly found in anaerobic environments (23). This supports the likely explanation that the bacterial community in the anaerobic earthworm gut migrates to soil environments and increases the carbon sequestration potential through CO_2_ fixation of the soil bacteria. In addition, the application of vermicompost increased the bacterial community richness compared to the control and swine manure compost-applied treatments (Fig. 1B). The increase in bacterial species richness promoted the utilization of more diverse carbon substrates, in turn increasing carbon use efficiency (CUE) (43). Previous studies have demonstrated the importance of CUE in predicting global SOC storage, and there is a positive correlation between microbial CUE and SOC storage in soil ecosystems (21). As a result, compared with compost application, the increased SOC storage under vermicompost application suggested that increased bacterial richness in vermicompost-applied soil could indirectly contribute to soil carbon sequestration by increasing the microbial CUE (44). In summary, vermicompost application promoted soil carbon sequestration by increasing the bacterial species richness and the abundance of carbon sequestration genes.

### Fewer virulent phages decreased bacterial carbon turnover in vermicompost-applied soil

Phage lytic infection is a key process in biogeochemical cycling, especially in soil nutrient turnover. However, the composition of the phage community in compost-applied soils is still poorly understood. The virulent phages could promote the turnover of bacterial communities and the release of organic matter taken up by bacteria into the environment (45, 46). Interestingly, we found that swine manure and vermicompost application had contrasting effects on the proportion of virulent phages in the soil. In contrast to the elevated proportion of virulent phages in swine manure-applied soil (92.8%), the proportion of virulent phages was significantly decreased, from 75.3% (control soil) to 36.9% in vermicompost-applied soil (Fig. S7). In addition, the host bacteria infected by virulent phages were less abundant in vermicompost-applied soil relative to swine manure compost-applied soil (Table S8), indicating that less bacterial organic matter was released into the environment via the lysis of host bacteria in vermicompost-applied soil. As a result, swine manure application likely increased while vermicompost application decreased the turnover of soil host bacterial organic carbon. However, soil virulent phage lysing host bacteria could lead to both soil carbon loss and carbon sequestration through the viral shunt and viral shuttle mechanisms (47). The variation in the proportion of virulent phages in SW and VE treatment was probably dependent on temperature changes during composting and the microbial community of the earthworm gut (48, 49). Overall, soil carbon loss/sequestration caused by virulent phages is still a “black box,” and more evidence is needed to quantify the contribution of phages to soil carbon turnover. In addition to DNA phages, RNA viruses also play an important role in soil carbon cycling via lysing host cells and encoding AMGs for carbon utilization, such as polygalacturonase 1 and cell wall hydrolase (8, 50, 51). In summary, soil RNA viruses may have an equally important impact on the soil carbon cycle as DNA phages, and there is an urgent need to strengthen the study of soil RNA viruses.

### Promising role of phage-encoded AMGs in soil carbon sequestration

The soil phage-encoded AMGs could affect bacterial metabolic activity through a bottom-up pathway, altering the ecological roles and competitive advantages of bacteria in the environment and regulating the cycling of soil elements (4). Soil phages encode AMGs related to the regulation of carbon metabolism, the degradation of soil organic matter, and the binding of polysaccharides as well as the activity of soil phage-carrying glycoside hydrolases (16, 18). To the best of our knowledge, this is the first study to investigate the roles of different types of manure application on the soil viromes, especially phage-mediated carbon metabolism in the soil. In accordance with previous studies, carbohydrate metabolism-associated AMGs were detected across all three treatments. The AMGs associated with carbon sequestration (e.g., GT6 and GT11) were detected across all three treatments. Notably, *GT6* and *GT11* were enriched in vermicompost-applied soil, and GT11 was identified for the first time in phage genomes (Fig. 4D). Both *GT6* and *GT11* are involved in the biosynthesis of lactosylceramide to glycosphingolipids, a long-chain hydrocarbon derivative of lipopolysaccharide that is essential for some bacterial cell membranes. This study observed bacteria-encoded glycosphingolipids across all three treatments (Table S4), including *Sphingomonas, Zymomonas mobilis*, and *Flectobacillus major* (52–57). Phage-encoded *GT6* and *GT11* contribute to carbon sequestration in microbial cells by transforming small-molecular lactosylceramide into more degradation-resistant large-molecular glycosphingolipids. Moreover, the upstream regions of both *GT6* and *GT11* in the phage genome contain promoters, increasing their expression possibilities (Fig. 5A). The exploration of promoter types (host promoters or phage promoters) that regulate phage AMG expression is a complex and interesting direction in the current field of phage biology research (58, 59). The different promoter types generate additional hypotheses, such as whether the phage life history/host range affects the AMGs transcriptional regulatory mechanisms (59). Addressing these hypotheses will enable fuller comprehension of the relationship between host/phage promoters and AMG expression, elevating the understanding of phage biology and its ecological impacts to new heights.

**Fig 6:**
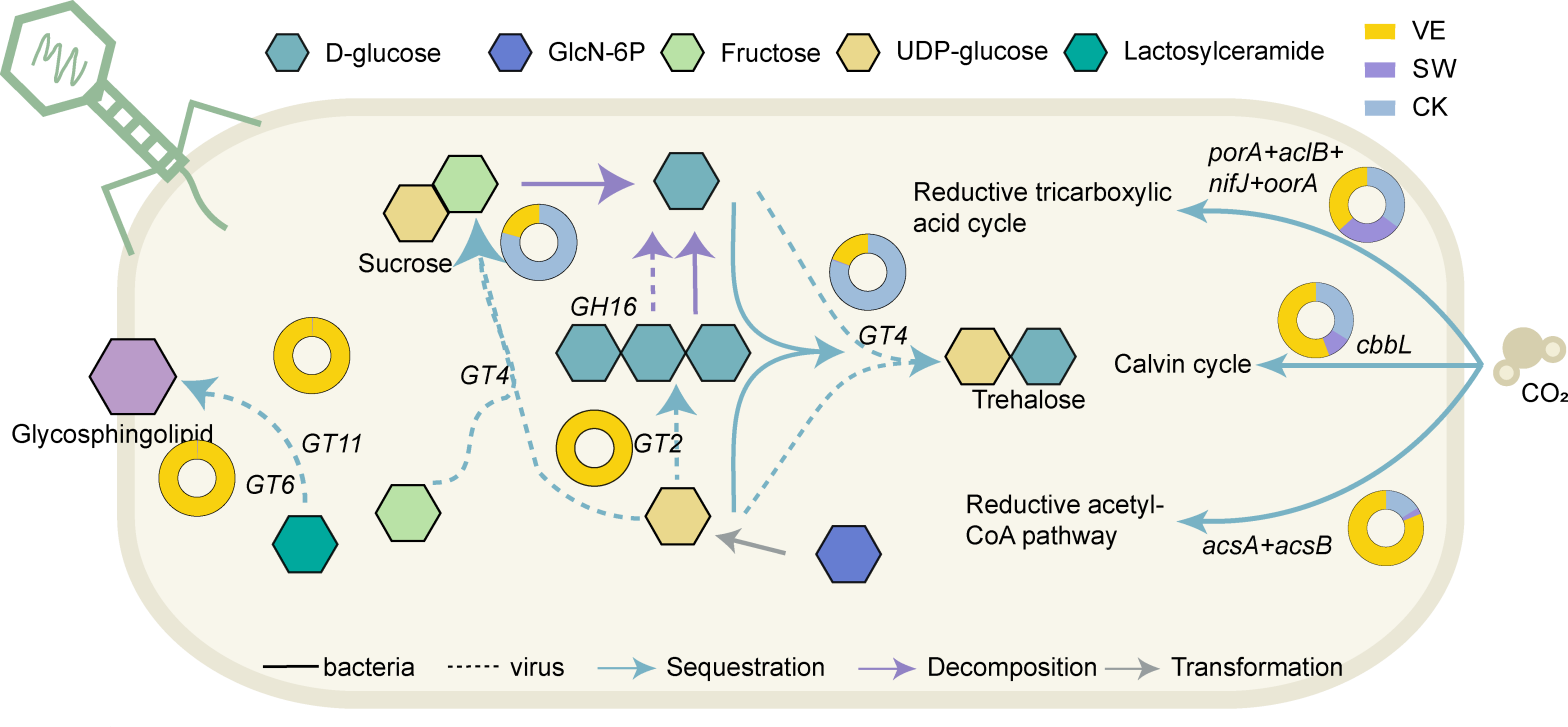
Overview of carbon metabolism pathway for virus-assisted host bacterial communities. The metabolic pathways of carbon sequestration, carbon transformation, and carbon decomposition with virus-bacteria interactions. The blue, gray, and purple arrows represent carbon sequestration, carbon transformation, and carbon decomposition processes, respectively. And the solid and dashed lines indicate the carbon metabolism processes participated in by bacteria and viruses, respectively. Different colors in the circles represent control (CK; blue), swine manure compost (SW; purple) and vermicompost (VE; yellow). The proportions of different colors in the circles were calculated based on the relative abundance of genes involved in the carbon metabolism process.

The contribution of phage AMGs to soil carbon sequestration in the different soil treatments was calculated. Vermicompost application clearly increased the carbon sequestration potential of phage AMGs compared to the other treatments. Notably, *GT*11 and *GT*6 comprised 100% coverage of glycosphingolipid synthesis genes in vermicompost-applied soils, suggesting that bacterial synthesis of glycosphingolipids was entirely dependent on the expression of phage-encoded AMGs. Moreover, the *GT*6 in vermicompost-applied soil was higher than in swine manure compost-applied soil and the control treatments, suggesting that the phage in vermicompost-applied soil could better assist the microbial hosts in synthesizing glycosphingolipids for soil carbon storage. The phage AMGs complemented the carbon sequestration pathways of fructose and UDP-glucose reacting to produce sucrose, and the alginate synthesis from UDP-glucose and ADP-glucose, compared to the purely bacterial community (Fig. 6). In addition, the AMGs *GT6* and *GT11* in the vermicompost-applied soil provided a novel carbon fixation pathway by participating in the synthesis of cell membrane glycosphingolipids in the host bacteria (Fig. 6). This led to the elevated carbon sequestration potential of phage AMGs in vermicompost-applied soil. The results indicate the potential of phages in vermicompost-applied soil to assist host bacteria in carbon fixation and to work with host bacteria in advancing the carbon cycling process. Our results provide a feasible basis for exploring the changes in the carbon sequestration capacity of soil microbial communities induced by phage AMGs. Future studies should quantify the effects of phages on global carbon cycle patterns at the scale of single cells, soil microbial communities, and ecosystems.

In this study, short-read sequencing was used to explore the role of phage AMGs in soil carbon sequestration. Although the positive role of phage AMGs was confirmed, due to the limitations of short-read sequencing technology, it was difficult to accurately trace carbon sequestration to specific phage species (60). As long-read sequencing technology can improve the integrity of phage genome assembly (60), the complementarity of the two sequencing technologies is expected to help in the accurate identification of phage species closely related to carbon sequestration and the in-depth analysis of their ecological niches, carbon sequestration patterns, and other metabolic capabilities. This would help to further clarify the micro-regulatory mechanism of phages on the carbon cycle in ecosystems, thereby promoting the development of carbon cycle research in the context of global climate change.

### Conclusions

In this study, we explored the carbon metabolism of the microbial communities in vermicompost and swine manure compost through metagenome and meta-virome sequencing analysis. We found that the application of the two types of compost played different roles in the carbon cycling process by altering the microbial community in different directions. Vermicompost application was associated with increased bacterial diversity, enriched phage-encoded AMGs involved in carbon sequestration, and elevated carbon sequestration compared to the control soil without fertilizer application. In contrast, swine manure application resulted in decreased bacterial diversity, more abundant phage AMGs associated with carbon decomposition, and a decrease in carbon sequestration potential compared to the control treatment. Notably, the enrichment of phage-encoded *GT*6 and *GT*11 in vermicompost soils was the two AMGs primarily linked to carbon sequestration, indicating their potential roles in assisting host bacteria to transform low-molecular-weight organic compounds into high-molecular-weight organic compounds that are highly resistant to degradation. In conclusion, our results have highlighted the importance of phage communities in vermicompost soils for bacteria-mediated carbon cycling in the soil. We demonstrated that phages in vermicompost soils could provide a novel tool for promoting soil carbon sequestration, and perhaps even mitigating climate change by providing important carbon sequestration-associated AMGs for their host bacteria.

## Data Availability

All raw sequence data generated in this research have been deposited in the NCBI’s Sequence Read Archive (SRA) database and can be accessed under project accession no. PRJNA1138505.

## SUPPLEMENTAL MATERIAL

Supplemental figures aem.00296-25-s0001.docx Figures S1 to S7.

Supplemental text aem.00296-25-s0002.docx Metagenome analysis of soil samples, and phage DNA extraction and virome analysis.

Supplemental tables aem.00296-25-s0003.xlsx Tables S1 to S12.

ASM does not own the copyrights to Supplemental Material that may be linked to, or accessed through, an article. The authors have granted ASM a non-exclusive, world-wide license to publish the Supplemental Material files. Please contact the corresponding author directly for reuse.
