# Supplementary material for: Expanding the potential soil carbon sink: unraveling carbon sequestration accessory genes in vermicompost phages
Source: Appl Environ Microbiol. 2025 Mar 14;91(4):e00296-25. doi: 10.1128/aem.00296-25 (PMC12016548; doi:10.1128/aem.00296-25)
Supplement: Supplemental figures — Figures S1 to S7. [file aem.00296-25-s0001.docx]

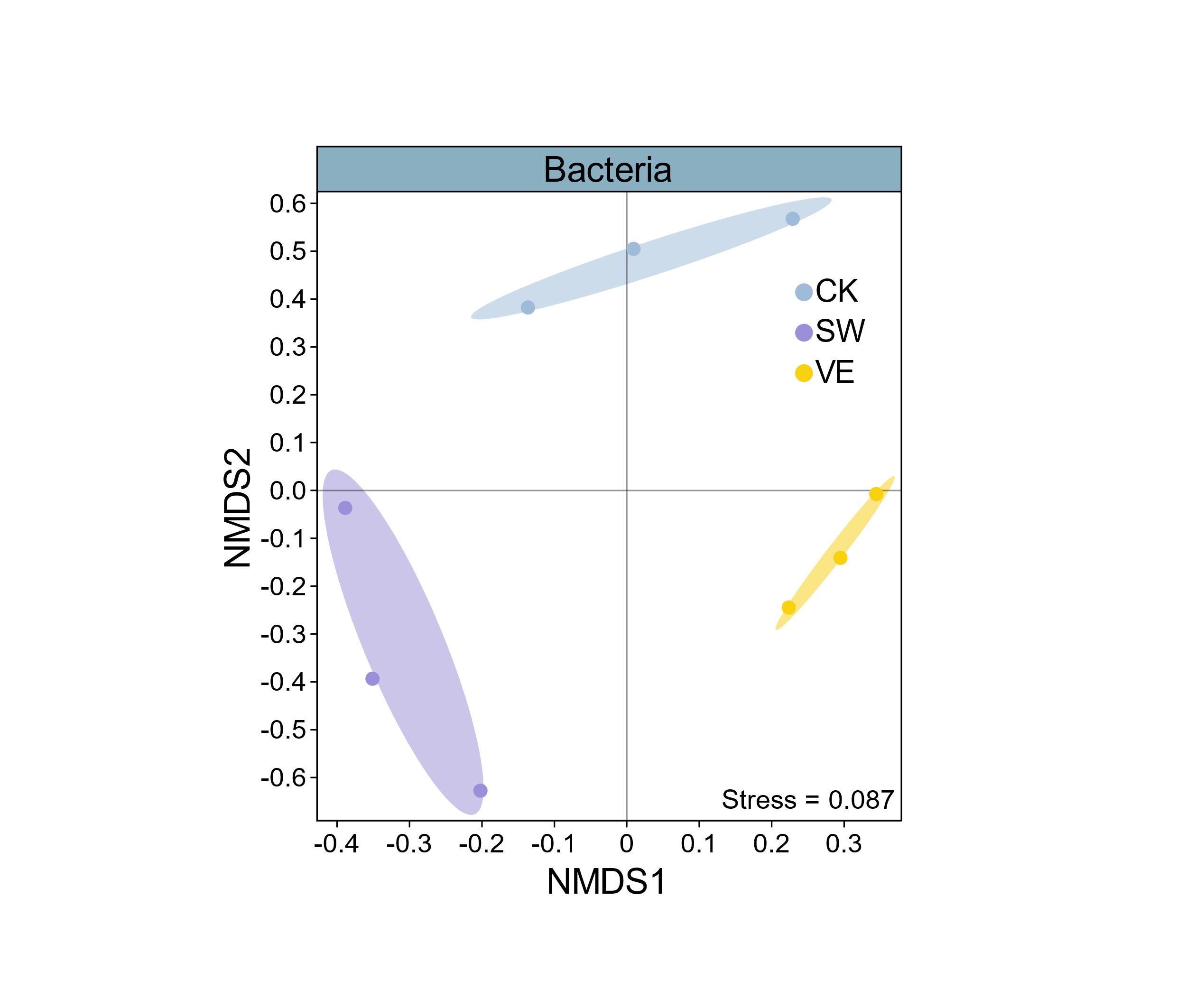


**Fig.S1** NMDS analysis of bacterial species in control (CK1-CK3) and composted soils (swine manure: SW1-SW3; vermicompost: VE1-VE3).
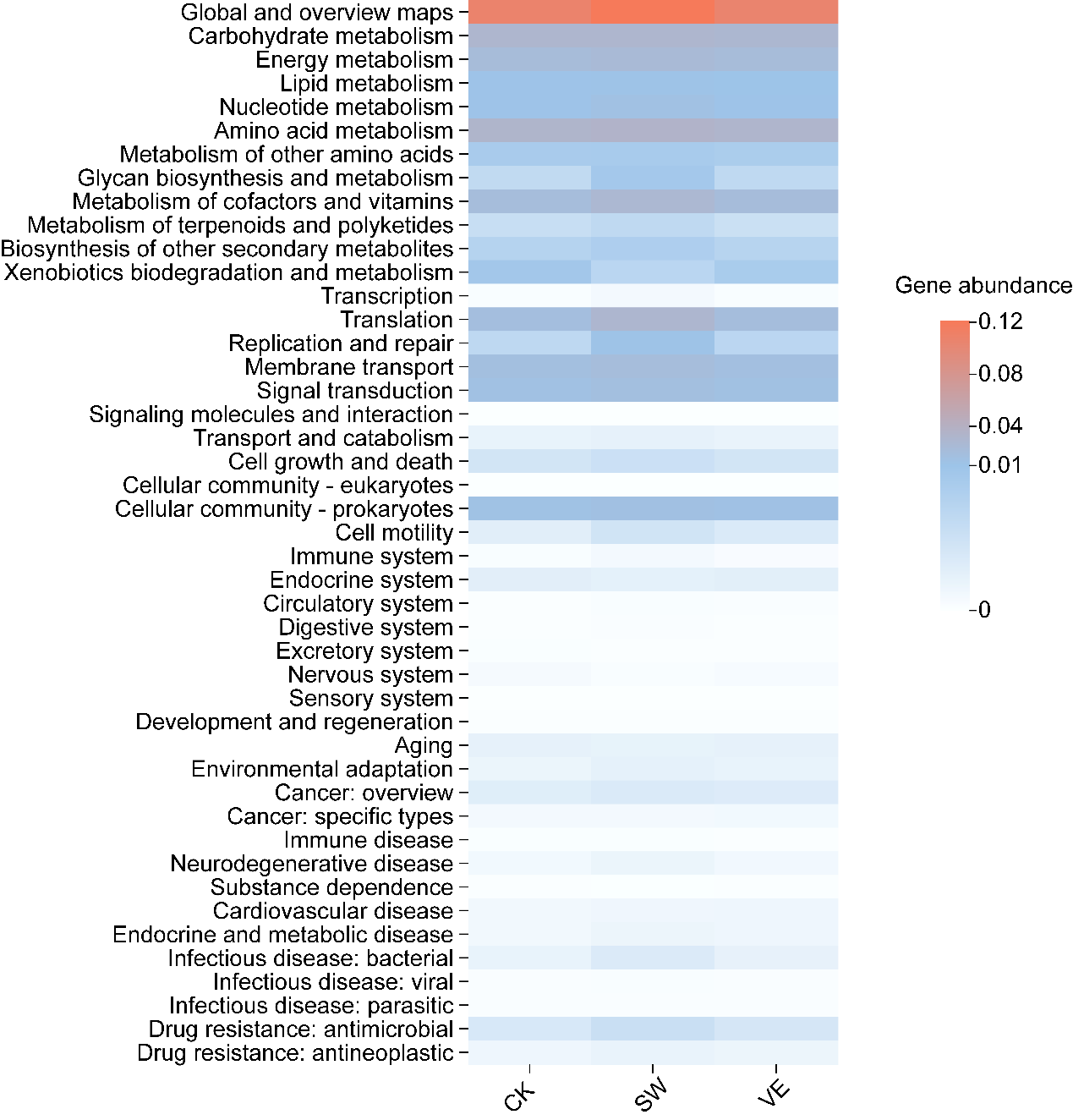


**Fig.S2** The heatmap shows the relative abundance of bacterial functional genes annotated by KEGG and CAZy databases in control (CK) and composted soils (swine manure: SW; vermicompost: VE).


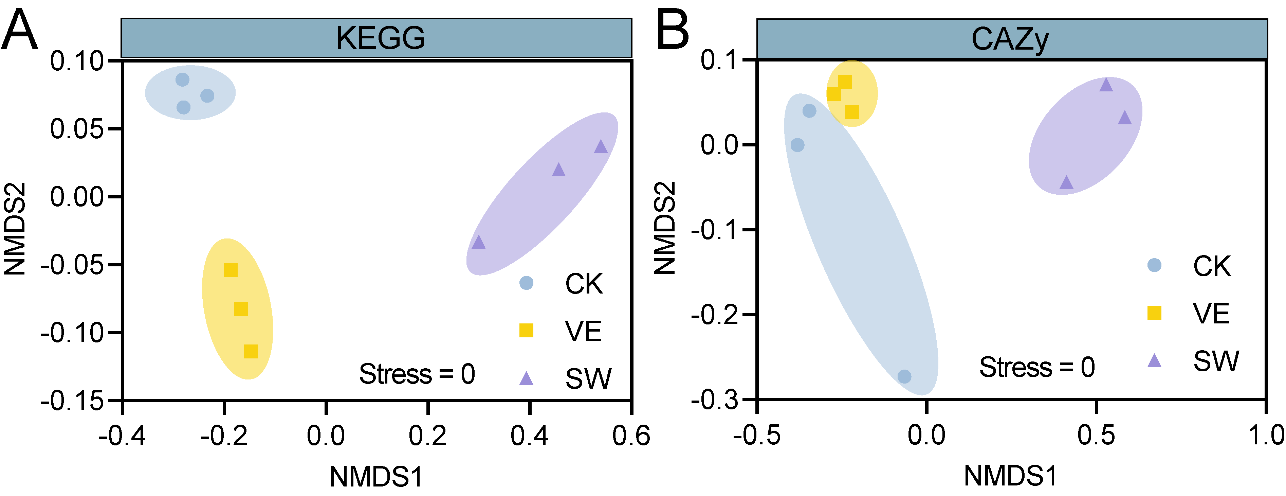


**Fig. S3** NMDS analysis of bacterial functional genes annotated by KEGG (**A**) and carbon-associated genes (**B**) in control (CK1-CK3) and composted soils (swine manure: SW1-SW3; vermicompost: VE1-VE3).


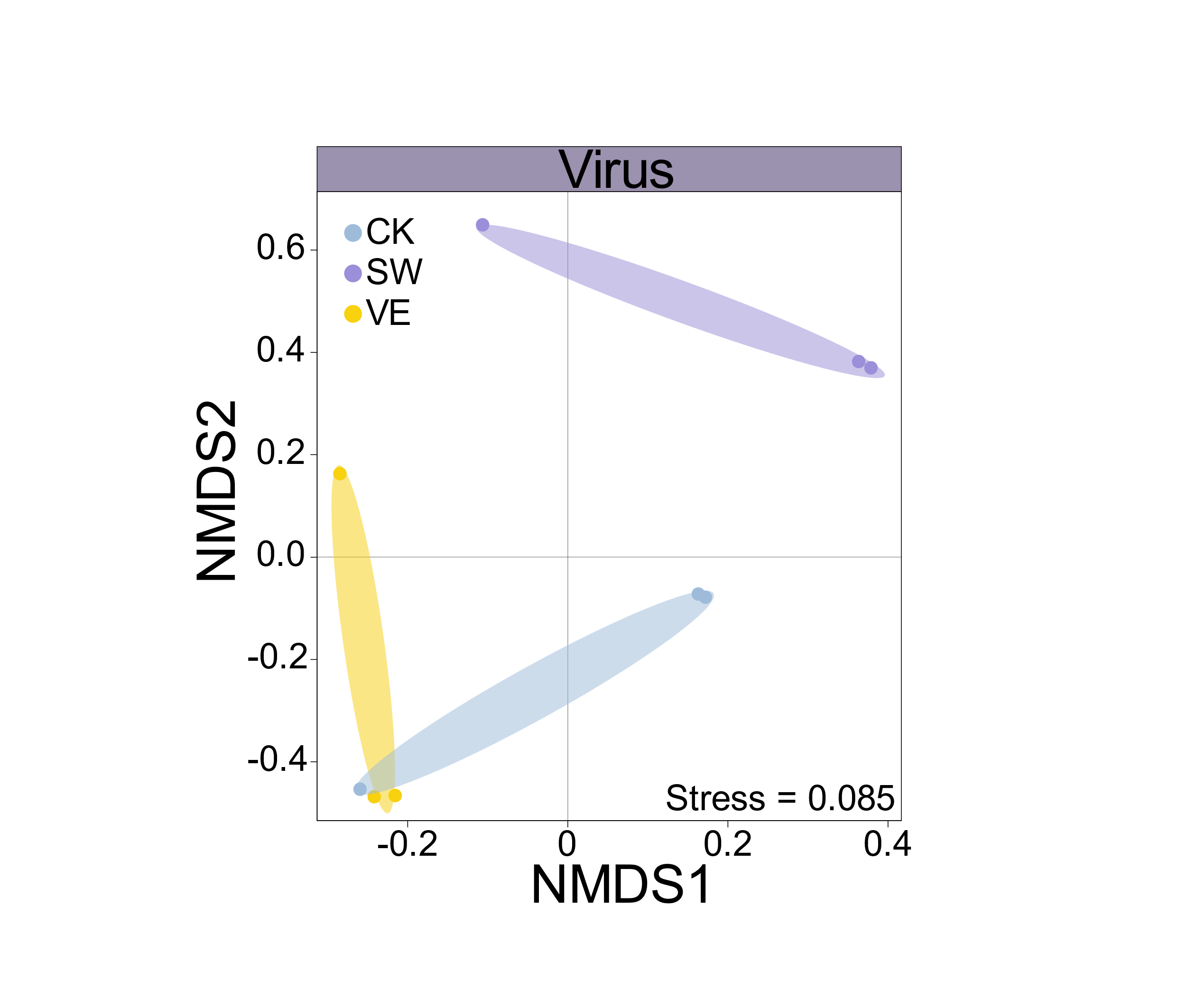


**Fig.S4** NMDS analysis of viral species in control (CK1-CK3) and composted soils (swine manure: SW1-SW3; vermicompost: VE1-VE3).


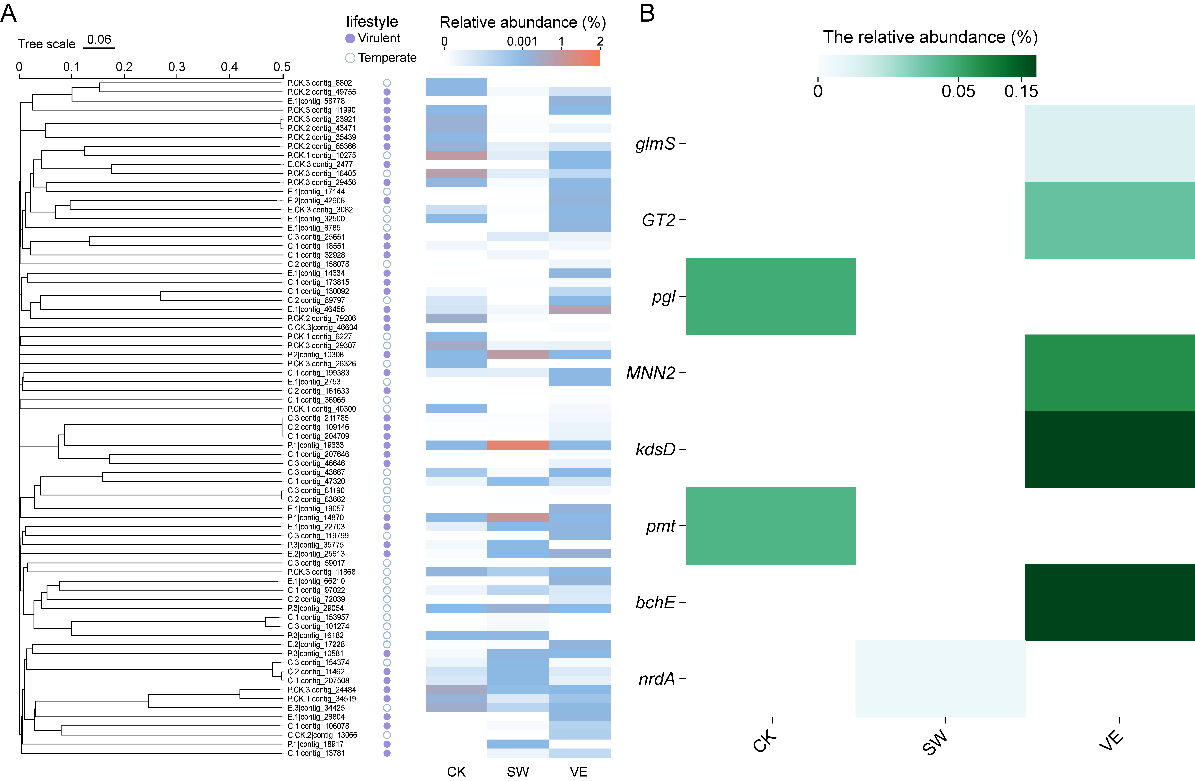


**Fig.S5 A:** The phylogeny of viruses containing AMGs, with purple and blank circles representing Virulent and Temperate viruses, respectively. The heatmap on the right represents the relative abundance of these viral contigs in control (CK) and composted soils (swine manure: SW; vermicompost: VE). **B:** The exclusive AMGs (i.e., absent in the other two treatments) in the in control (CK) and composted soils (swine manure: SW; vermicompost: VE).


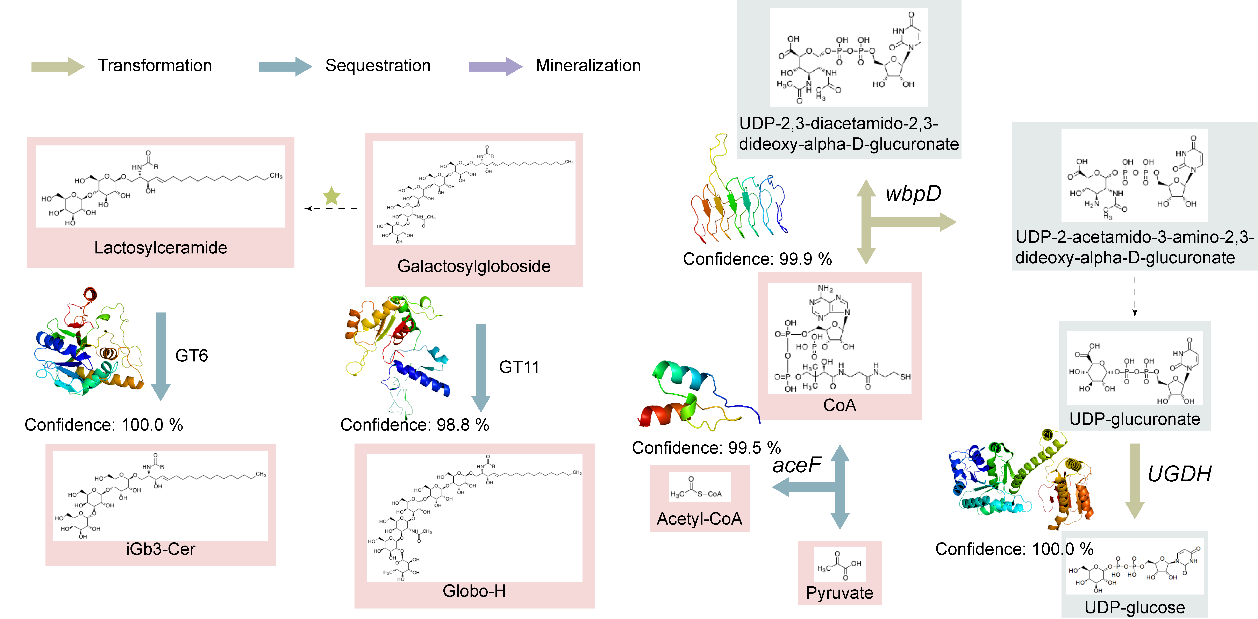


**Fig.S6** The action pathways and metabolites of the five carbon sequestration-associated AMGs (*GT6*, *GT11*, *aceF*, *wbpD*, and *UGDH*), with different colored arrows representing the presence of AMGs in the different treatments.


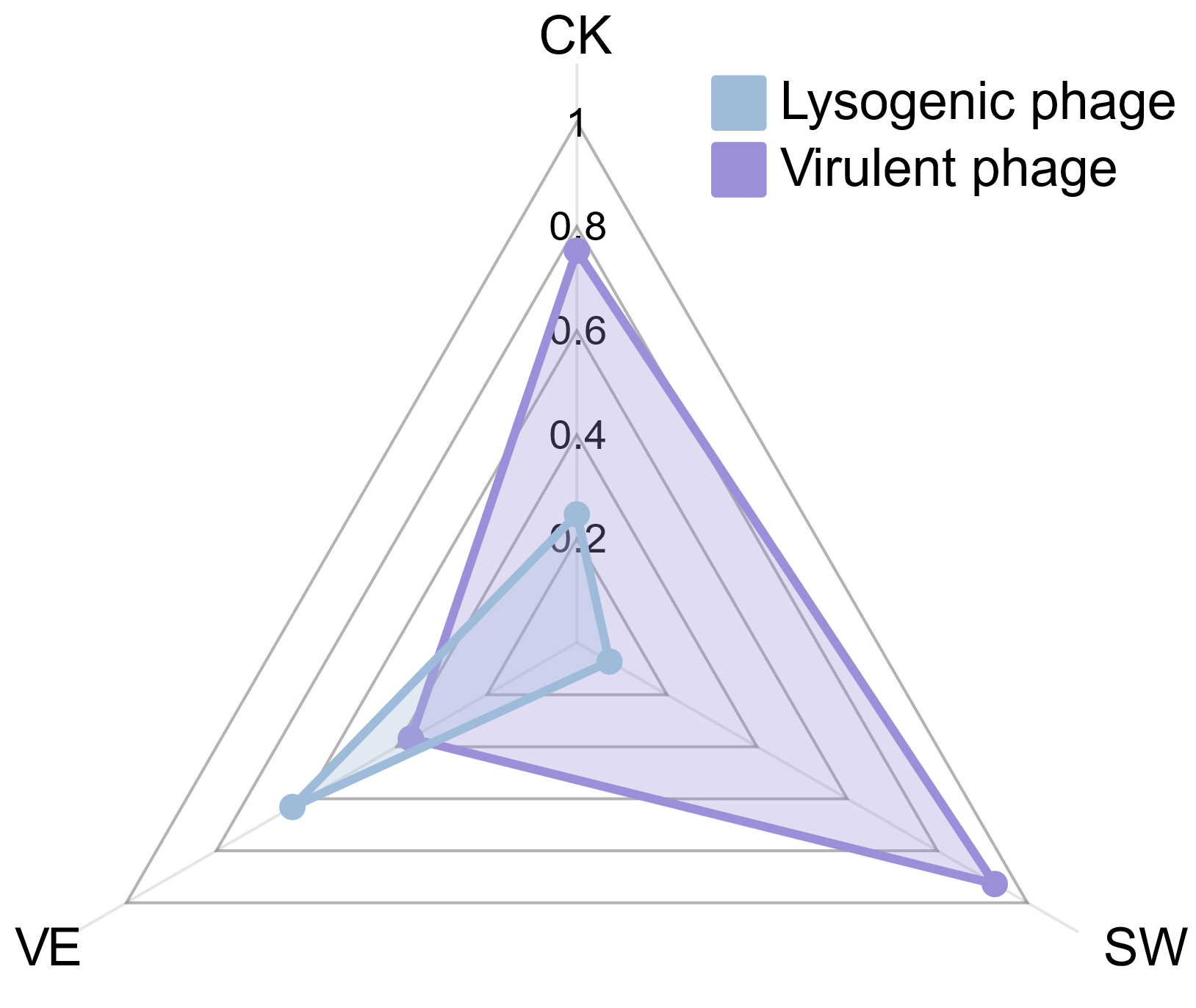


**Fig.S7** The proportion of virulent and lysogenic phages in control (CK) and composted soils (swine manure: SW; vermicompost: VE).
