# Supplementary material for: Expanding the potential soil carbon sink: unraveling carbon sequestration accessory genes in vermicompost phages
Source: Appl Environ Microbiol. 2025 Mar 14;91(4):e00296-25. doi: 10.1128/aem.00296-25 (PMC12016548; doi:10.1128/aem.00296-25)
Supplement: Supplemental text — Metagenome analysis of soil samples, and phage DNA extraction and virome analysis. [file aem.00296-25-s0002.docx]

**Metagenome analysis of soil samples**

The total DNA from all fresh soil samples (0.5 g) was extracted using a FastDNA Spin kit for soil (MP Biomedicals, CA, USA) following the manufacturer’s instructions. The purity and concentration of total DNA was identified by using a Life Technologies Qubit 4.0 (Supplementary Table 2). The DNA concentration were 27.6±3.5b, 73.6±14.3a, 63.0±4.3a ng μL-1 (t-test) for CK, SW, and VE treatments, respectively. Sequencing libraries were conducted using an NEB Next® Ultra™ DNA Library Prep Kit for Illumina® (New England Biolabs, MA, USA) following the manufacturer's instructions. The library quality was assessed by using a Qubit® dsDNA HS Assay Kit (Life Technologies, Grand Island, NY) and an Agilent 4200 (Agilent, Santa Clara, CA) system. Nine libraries were constructed for whole-genome shotgun approach, and paired-end (PE, 2 × 150 bp) sequencing was carried out on an Illumina HiSeq 2500 high-throughput sequencing platform. After quality control of the raw data with Cutadapt (v 1.2.1), a total of ~0.55 billion clean reads (~59.06 million per CK treatment, ~61.54 million per SW treatment, and ~62.23 million per VE treatment; Supplementary Table 3) and of average target clean bases of 8.4 Gbp per metagenome were obtained and used for *de novo* assembly by using MEGAHIT (--k-min 35 --k-max 95 --k-step 20 --min-contig-len 500 -m 0.1) (<https://github.com/voutcn/megahit>)(1). Then, the open reading frame (ORF) was predicted by MetaGeneMark (<http://exon.gatech.edu/GeneMark/metagenome>), the CD-HIT (v4.8.1) was used to remove redundancy genes with the parameters”-aS 0.90 -c 0.90”, and the corresponding unigenes data set was obtained(2). The non-redundant unigenes sequences were compared against the NCBI-NR database for BLAST comparison of species using Diamond (with a threshold set at e-value ≤ 0.0001). Since each unigenes may have multiple comparison results and different classification levels, the lowest common ancestor (LCA) algorithm was adopted, and the classification level before the first branch appeared was used as the species annotation information of the unigenes (Supplementary Table 4)(3). The microbial taxonomic alpha and beta diversity analyses, including alpha indexes (Chao1, Richness, Shannon, and Pielou index) and non-metric multidimensional scaling (NMDS) were conducted using the vegan and ggplot2 packages in R(4).

Then, unigenes were mapped to the clean data using “bwa mem” from BWA (v0.7.17, -k 30) with default parameters to determine the Reads Per Kilobase per Million mapped reads (RPKM) of genes in each sample. The relative abundance of genes was calculated by dividing the RPKM of each gene in each sample by the sum of the RPKM of all genes. Functional genes were annotated by matching to the KEGG database (Release 101.0) and eggNOG (v4.5.1) using DIAMOND with an *e*-value ≤ 0.001 (Supplementary Table 5)(4). Notably, genes associated with carbon metabolism were further identified based on the CAZy database (cazydb .07312018.fa), and classified into three different categories, including carbon sequestration, carbon transformation, and carbon decomposition (Supplementary Table 6)(5).

**Phage DNA extraction and virome analysis**

The total soil phages (both the free phages and the prophages) DNA were extracted following the method described by Zheng et al.(6) and Tang et al.(7) with following modifications. Firstly, the soil samples (500 g) were homogenized through 0.25 mm-sieve, and were mixed with 500 mL of 1% (*w/w*) [potassium citrate](https://www.sciencedirect.com/topics/biochemistry-genetics-and-molecular-biology/potassium-citrate) buffer (10 g·L^-1^ C_6_H_5_K_3_O_7_, 1.92 g·L^-1^ Na_2_HPO_4_·12H_2_O, 0.24 g·L^-1^ KH_2_PO_4_, pH = 7), incubated at 4 °C for 15 min, then sonicated (100 W, 47 kHz) in an ice bath for 3 min. During the above-described procedure, each extractant was shaken for 30 s after 1 min of ultrasound. The supernatant was obtained via [centrifugation](https://www.sciencedirect.com/topics/biochemistry-genetics-and-molecular-biology/centrifugation) at 7000 rpm for 10 min (this step was repeated twice) and was then filtered sequentially through 0.45 μm and 0.22 μm sterilized filters (PTFE, hydrophilic phase) to remove non-phage particles. Next, the supernatant was concentrated using a tangential flow filtration system (TFF; Sartorius Vivaflow50 30000MWCO PES, USA; 100 kDa). The free phage suspension was enriched by the TFF system. Secondly, the induction of prophages was performed by mixing soil slurry (each soil sample was suspended in deionized water) and mitomycin-C solution at a final concentration of 1 μg mL^-1^. After incubated at room temperature, with shaking at 200 rpm for 24 h in the dark, the culture was concentrated following the above procedures to harvest the induced free phages solution. No contamination of bacterial DNA was found in the mixture of free phage enrichment solution and the induced free phages enrichment solution as examined by 16S rRNA gene PCR analysis. A Takara MiniBEST Viral RNA/DNA Extraction Kit 5.0 was used to extract the total phage DNA from the mixture of free phage enrichment solution and the induced free phages enrichment solution following the manufacturer’s instructions. Then, the whole genome was amplified by using a Qiagen kit (150054 REPLI-g Cell WGA & WTA Kit), and a Thermo NanoDrop One, a Life Technologies Qubit 4.0, and 1.5% agarose electrophoresis were used to analyze the amplification products. Sequencing libraries were generated using an NEB Next® Ultra™ DNA Library Prep Kit for Illumina® (New England Biolabs, MA, USA) following the manufacturer's instructions. The library quality was assessed by using a Qubit® dsDNA HS Assay Kit (Life Technologies, Grand Island, NY) and an Agilent 4200 (Agilent, Santa Clara, CA) system. Finally, the library was sequenced on an Illumina Novaseq 6000 platform, and 150 bp paired-end reads were generated. After quality control of the raw data with Cutadapt (v 1.2.1), a total of ~0.27 billion clean reads (~31.99 million per CK treatment, ~31.57 million per SW treatment, and ~27.80 million per VE treatment) were obtained (Supplementary Table 3).

All the clean reads were used for *de novo* co-assembly of phage sequences to address potential viromes among three treatments using Megahit with k-mer ~ parameter setting. The assembled contigs longer than 5000bp were defined as phages if they met one of the following criteria. First, viral-specific k-mer patterns were checked by DeepVirFinder (default parameters)(8). Contigs with scores ≤ 0.9 and *p*-values < 0.05 were excluded from further analysis. Second, VirSorter2 was employed for phage identification with the criterion of a score ≥ 0.9(9). Finally, based on the benchmark evaluation of these two tools, CheckV was used for ultimate verification(10). vConTACT2 (v.2.0) was used to analyze the protein-sharing network of the phage contigs(11). The non-redundant phage sequnences were clustered with the NCBI RefSeq database and the permafrost soil phage database based on protein similarity. Briefly, the vOTU protein sequences were grouped into PCs (protein clusters) via blastp using default parameters. The similarity between vOTUs was calculated based on the number of shared PCs. Then, pairs of vOTUs that had a degree of similarity ≥ 1 were grouped into phage clusters. Finally, Cytoscape (v 3.9.0) was used to visualize the shared network(12). Meanwhile, we used PhaGCN2.0 to annotate the specific phage taxon according to the latest ICTV classification tables with the parameter “--len 5000” (Supplementary Table 7)(13). The viromes clean reads were mapped to the phage contigs using “bwa mem” from BWA (v0.7.17, -k 30) with default parameters to determine the RPKM of phage contigs in each sample. The relative abundance of phage contigs was calculated by dividing the RPKM of each contig in each sample by the sum of the RPKM of all contigs (14) (Supplementary Table 7). The lifestyle of each phage contig (i.e., virulent or temperate phage) was predicted by Deephage (Supplementary Table 7)(15). A phage phylogeny was constructed by using ViPTree v4.0 with the only query model (16). The final phylogenetic tree was visualized using Chiplot(17). We selected the top ten phage families except for those that were unclassified with the highest phage relative abundances for visualization, and calculated diversity (including alpha indexes Chao1, Richness, Shannon, and Pielou and NMDS analyze) using the vegan and ggplot2 packages in R.

Reference:

1. Li D, Luo R, Liu C-M, Leung C-M, Ting H-F, Sadakane K, Yamashita H, Lam T-W. 2016. MEGAHIT v1.0: A fast and scalable metagenome assembler driven by advanced methodologies and community practices. Methods 102:3–11.

2. Fu L, Niu B, Zhu Z, Wu S, Li W. 2012. CD-HIT: accelerated for clustering the next-generation sequencing data. Bioinformatics 28:3150–3152.

3. Blanco-Míguez A, Beghini F, Cumbo F, McIver LJ, Thompson KN, Zolfo M, Manghi P, Dubois L, Huang KD, Thomas AM, Nickols WA, Piccinno G, Piperni E, Punčochář M, Valles-Colomer M, Tett A, Giordano F, Davies R, Wolf J, Berry SE, Spector TD, Franzosa EA, Pasolli E, Asnicar F, Huttenhower C, Segata N. 2023. Extending and improving metagenomic taxonomic profiling with uncharacterized species using MetaPhlAn 4. Nat Biotechnol 41:1633–1644.

4. Yuan S, Friman V-P, Balcazar JL, Zheng X, Ye M, Sun M, Hu F. 2023. Viral and Bacterial Communities Collaborate through Complementary Assembly Processes in Soil to Survive Organochlorine Contamination. Applied and Environmental Microbiology 89:e01810-22.

5. Dai Z, Zang H, Chen J, Fu Y, Wang X, Liu H, Shen C, Wang J, Kuzyakov Y, Becker JN, Hemp A, Barberán A, Gunina A, Chen H, Luo Y, Xu J. 2021. Metagenomic insights into soil microbial communities involved in carbon cycling along an elevation climosequences. Environ Microbiol 23:4631–4645.

6. Zheng X, Jahn MT, Sun M, Friman V-P, Balcazar JL, Wang J, Shi Y, Gong X, Hu F, Zhu Y-G. 2022. Organochlorine contamination enriches virus-encoded metabolism and pesticide degradation associated auxiliary genes in soil microbiomes. ISME J 16:1397–1408.

7. Tang X, Zhong L, Tang L, Fan C, Zhang B, Wang M, Dong H, Zhou C, Rensing C, Zhou S, Zeng G. 2023. Lysogenic bacteriophages encoding arsenic resistance determinants promote bacterial community adaptation to arsenic toxicity. ISME J 17:1104–1115.

8. Ren J, Song K, Deng C, Ahlgren NA, Fuhrman JA, Li Y, Xie X, Poplin R, Sun F. 2020. Identifying viruses from metagenomic data using deep learning. Quant Biol 8:64–77.

9. Guo J, Bolduc B, Zayed AA, Varsani A, Dominguez-Huerta G, Delmont TO, Pratama AA, Gazitúa MC, Vik D, Sullivan MB, Roux S. 2021. VirSorter2: a multi-classifier, expert-guided approach to detect diverse DNA and RNA viruses. Microbiome 9:37.

10. Nayfach S, Camargo AP, Schulz F, Eloe-Fadrosh E, Roux S, Kyrpides NC. 2021. CheckV assesses the quality and completeness of metagenome-assembled viral genomes. Nat Biotechnol 39:578–585.

11. Bin Jang H, Bolduc B, Zablocki O, Kuhn JH, Roux S, Adriaenssens EM, Brister JR, Kropinski AM, Krupovic M, Lavigne R, Turner D, Sullivan MB. 2019. Taxonomic assignment of uncultivated prokaryotic virus genomes is enabled by gene-sharing networks. Nat Biotechnol 37:632–639.

12. Shannon P, Markiel A, Ozier O, Baliga NS, Wang JT, Ramage D, Amin N, Schwikowski B, Ideker T. 2003. Cytoscape: a software environment for integrated models of biomolecular interaction networks. Genome Res 13:2498–2504.

13. Jiang J, Yuan W, Shang J, Shi Y, Yang L, Liu M, Zhu P, Jin T, Sun Y, Yuan L-H. 2023. Virus classification for viral genomic fragments using PhaGCN2. Brief Bioinform 24:bbac505.

14. Li H, Durbin R. 2009. Fast and accurate short read alignment with Burrows-Wheeler transform. Bioinformatics 25:1754–1760.

15. Wu S, Fang Z, Tan J, Li M, Wang C, Guo Q, Xu C, Jiang X, Zhu H. 2021. DeePhage: distinguishing virulent and temperate phage-derived sequences in metavirome data with a deep learning approach. Gigascience 10:giab056.

16. Nishimura Y, Yoshida T, Kuronishi M, Uehara H, Ogata H, Goto S. 2017. ViPTree: the viral proteomic tree server. Bioinformatics 33:2379–2380.

17. Xie J, Chen Y, Cai G, Cai R, Hu Z, Wang H. 2023. Tree Visualization By One Table (tvBOT): a web application for visualizing, modifying and annotating phylogenetic trees. Nucleic Acids Res 51:W587–W592.
